# Functional differentiation and genetic diversity of rice cation exchanger (CAX) genes and their potential use in rice improvement

**DOI:** 10.1038/s41598-024-58224-2

**Published:** 2024-04-15

**Authors:** Shangshu Lian, Yanjun Chen, Yanyan Zhou, Ting Feng, Jingsi Chen, Lunping Liang, Yingzhi Qian, Tao Huang, Chenyang Zhang, Fengcai Wu, Wenli Zou, Zhikang Li, Lijun Meng, Min Li

**Affiliations:** 1 School of Agronomy, Anhui Agricultural University, Hefei, 230036 China; 2 Shenzhen Branch, Guangdong Laboratory of Lingnan Modern Agriculture, Genome Analysis Laboratory of the Ministry of Agriculture and Rural Affairs, Agricultural Genomics Institute at Shenzhen, Chinese Academy of Agricultural Sciences, Shenzhen, 518120 China

**Keywords:** Agricultural genetics, Genomics

## Abstract

Cation exchanger (CAX) genes play an important role in plant growth/development and response to biotic and abiotic stresses. Here, we tried to obtain important information on the functionalities and phenotypic effects of CAX gene family by systematic analyses of their expression patterns, genetic diversity (gene CDS haplotypes, structural variations, gene presence/absence variations) in 3010 rice genomes and nine parents of 496 Huanghuazhan introgression lines, the frequency shifts of the predominant gcHaps at these loci to artificial selection during modern breeding, and their association with tolerances to several abiotic stresses. Significant amounts of variation also exist in the cis-regulatory elements (CREs) of the OsCAX gene promoters in 50 high-quality rice genomes. The functional differentiation of OsCAX gene family were reflected primarily by their tissue and development specific expression patterns and in varied responses to different treatments, by unique sets of CREs in their promoters and their associations with specific agronomic traits/abiotic stress tolerances. Our results indicated that *OsCAX1a* and *OsCAX2* as general signal transporters were in many processes of rice growth/development and responses to diverse environments, but they might be of less value in rice improvement. *OsCAX1b*, *OsCAX1c*, *OsCAX3* and *OsCAX4* was expected to be of potential value in rice improvement because of their associations with specific traits, responsiveness to specific abiotic stresses or phytohormones, and relatively high gcHap and CRE diversity. Our strategy was demonstrated to be highly efficient to obtain important genetic information on genes/alleles of specific gene family and can be used to systematically characterize the other rice gene families.

## Introduction

In a plant species with a wide geographic distribution, different members of a gene family may often become functionally differentiated. This functional differentiation among different members of a gene family, reflected by their sequence diversity among related species, as well as among different populations of a species, is of great interest to plant scientists because it is the consequences of the plant species or populations adapted to different ecological environments. Thus, dissecting the genetic diversity of different members of a gene family in a plant species is expected to provide important information on their functionalities and, thus, their potential uses in plant improvement.

Calcium (Ca^2+^) serves as a widespread secondary messenger in plant cells, facilitating a range of intricate responses and regulatory mechanisms in plants in response to biotic and abiotic stress through signal transduction^1^. Changes in Ca^2+^ concentration within the plant cytoplasm are crucial for signal transduction. Stimulation of plants by the external environment leads to rapid fluctuations in cytoplasmic Ca^2+^ levels, triggering a response of calcium signaling channels^2–6^. This process is achieved through interactions between numerous channel proteins and transport proteins on the cell membrane, which in turn regulate gene expression and various physiological reactions in plants^7^. Various signals are initiated by different Ca^2+^ sensors, including calmodulin (CaM), CaM-like proteins (CMLs), and calcineurin B-like proteins (CBLs), in order to elicit specific and tailored responses, thereby regulating gene expression and metabolism in plants^8^. Among these Ca^2+^ sensors, the cation exchanger (CAX) gene family has garnered significant attention. CAX represents a subset of the CaCA (Ca^2+^/Cation antiporter) superfamily, primarily situated in the vacuolar membrane^9^. Its function involves the of cellular cation homeostasis by transporting cytoplasmic cations, such as calcium, into vesicular storage using the pH gradient generated by the proton pump^10,11^. Strong evidence from Arabidopsis and other plants indicates that CAX genes play a crucial role in polycation tolerance, metal transport, element distribution, ion homeostasis, and abundance in response to abiotic stress^10,12^. In these processes, CAX genes function to restore Ca^2+^ in both intracellular and extracellular reservoirs of the cytoplasm, as well as distribute Ca^2+^ into plant vacuoles for continual signal perception, ensuring the progress of various biochemical reactions by interacting with Ca^2+^ channels^9,11,13,14^. For instance, in Arabidopsis, *AtCAX1* expression is significantly induced by cold stress, while the *CAX2* mutant exhibits flooding tolerance^15^. Additionally, in rice, *OsCAX1a*, *OsCAX1c*, and *OsCAX4* may be involved in the absorption and transport of cadmium^15,16^, with *OsCAX1a* and *OsCAX3* conferring tolerance to manganese^17^. The CAX gene families in different plant species vary considerably in number and sequence due to their diverse polyploid histories during evolution^18–20^. Therefore, understanding the functional differentiation of duplicated members of the CAX gene family and their genetic diversity within and among different populations has been a subject of widespread interest.

As one of the most important food crops, rice is grown in diverse ecologies worldwide and often faces a wide range of biotic and abiotic stress. Rice is also a model plant species for functional and population genomic research, with more than 4600 rice genes cloned and functionally characterized at the molecular level **(**https://funricegenes.github.io/)^21,22^. The past few decades have also witnessed tremendous progress in the genetic and molecular dissection of complex traits of rice using DNA marker-facilitated genetic mapping and various omics tools, such as transcriptomic, proteomic, and metabolomic analyses, generating huge amounts of data on the genetic and molecular mechanisms underlying complex traits and responses to biotic and abiotic stress in rice. To facilitate the integration of research advances in rice functional genomic research for rice improvement, rice population genomic research has proceeded more recently at an unprecedented rate. To date, more than 10,000 rice germplasm accessions worldwide have been re-sequenced^23–25^. Rice population genomic research has revealed tremendous amounts of genomic diversity in rice germplasm accessions and their organization within and among different rice populations, including almost unlimited numbers of single nucleotide polymorphisms (SNPs), huge numbers of structural variations (SVs) of > 30 bps, gene presence/absence variations (PAVs) for about 50% of rice genes, extremely rich gene-coding sequence (CDS) haplotype (gcHap) diversity in most rice genes, and gene copy number variation (CNVs) for a significant portion of rice genes^23,26–29^. One of the exciting discoveries from the 3010 rice genomes project (3KRGP) was the impact of modern breeding on the gcHap diversity of 45,963 rice genes, reflected primarily by changes in gcHap diversity and frequency shifts of the predominant (ancient) alleles in modern rice varieties compared to the landraces of two rice subspecies resulting from artificial selection during modern breeding^27^. In contrast to most SNPs in the inter-genic regions and introns of genes, gene PAVs, CNVs, and SVs that occur in genic regions and major gcHaps are expected to have large phenotypic effects and thus are powerful markers for genetic and molecular dissection of complex traits, as demonstrated more recently in the cases of *Sub1a GL7* and *qSW5*^30–32^. Using a rice super pan‐genome, natural allelic variation in *TTL1* has been shown to control thermotolerance and grain size^33^. Several large and comprehensive databases have been established from rice functional and population genomic studies and have been made publicly available to all researchers^34^. However, it remains a great challenge to integrate the advances and huge amounts of information from rice functional and population genomic research into more efficient strategies for rice improvement. This is particularly true for > 90% rice genes with their functions unknown and the presence of many functional but uncharacterized alleles in each of the rice genes. Thus, one important question arises regarding how to obtain essential information on gene functionality and phenotypic effects more efficiently without going through the time-consuming efforts of the classical gene cloning process, given the presence of so much genetic and molecular information on rice genes in the public domain.

In this study, we reported an effort in functional characterization and allelic mining of the rice CAX gene family containing six members (*OsCAX1a*, *OsCAX1b*, *OsCAX1c*, *OsCAX2*, *OsCAX3*, and *OsCAX4*) by systematic analyses of their homology relationships among different plant species, cis-regulatory element (CRE) variation in promoter regions, expression patterns, genetic diversity in different rice populations, and associations with tolerance to different abiotic stress using publicly available data. We also analyzed their associations with salt tolerance using 3KRGP and eight introgression line (IL) populations. Our results demonstrated a powerful and highly efficient strategy for obtaining important genetic information on rice genes of unknown function essential for improving abiotic stress tolerance in rice using breeding by design in the future.

## Materials and methods

### Homology analysis, protein structure and domain prediction of CAX genes in different species

Six rice CAX genes (*OsCAX1a, OsCAX1b, OsCAX1c, OsCAX2, OsCAX3,* and *OsCAX4*) from the CAX gene family were examined in this study. Among these genes, only *OsCAX1a* was reported to be functionally related to rice panicle degeneration, while the functions of the other five rice CAX genes remain unknown^35,36^. To investigate the relationships between CAX genes in different species, the sequences of the six rice CAX genes were compared with those of *Arabidopsis thaliana* (*At*), *Triticum aestivum* (*Ta*), *Zea mays* (*Zm*), *Glycine max* (*Glyma*), *Hordeum vulgare* (*Hv*), *Solanum tuberosum* (*St*), *Solanum lycopersicum* (*Sl*), *Saccharomyces cerevisiae* (*Sc*), *Synechococcus sp* (*Ssp*), and *Escherichia coli* (*Ec*). Phylogenetic analysis of the CAX genes in these species was conducted using MEGA X software. The protein sequences of the CAX genes in the aforementioned species were compared using a logarithmic expected multi-sequence comparison method called CDS DNA sequence comparison. Names and the protein sequences of 62 CAX genes were listed in Table S1. Phylogenetic trees based on full-length protein sequence alignment were constructed using the neighbor-joining (NJ) method with 1000 bootstrap replicates. The CAXs protein structure and domain was predicted on https://swissmodel.expasy.org/ and https://www.genome.jp/^37^. Number of motifs were listed in Table S2.

### Collinearity relationship and promoter cis-regulatory element analysis of the OsCAX gene family

Gene duplication patterns and collinearity relationships of the OsCAX gene family were identified and analyzed using MCScanX^38^. The ratio of synonymous mutations to non-synonymous mutations (Ka/Ks) between gene pairs was calculated using TBtools^39^ to determine the evolutionary selection pressure on each member of the OsCAX gene family. The 2000 bp sequences upstream of the translation start sites of each OsCAX gene were retrieved from the Os-Nipponbare-Reference-IRGSP-1.0 reference genome as the promoter sequence where the cis-regulatory elements were predicted using PlantCARE (Table S3) (http://bioinformatics.psb.ugent.be/webtools/plantcare)^40^. The results were visualized using TBtools. We also selected 25 *Xian* (*indica*) and 25 *Geng* (*japonica*) accessions from 33 and 111 high-quality rice genomes^26,29^. The 50 high-quality accessions information are available in the Table S4. We used “blastp” with setting the alignment threshold to 1e^-10^, to align and compare the CAX protein sequences of the Nipponbare reference genome with those of 50 samples. Subsequently, we used a Perl script to extract the promoter sequences 2000 bp upstream of each of the CAX genes and analyzed their variation in the predicted type and number/frequency of CREs using PlantCARE and a shell script.

### Expression profile analysis of the rice CAX genes

We downloaded the expression profiles of the six rice CAX genes from three rice expression databases: one was built from the *Geng* variety Nipponbare (http://expression.ic4r.org/) (https://www.mbkbase.org/rice)^41,42^, and the other from two *Xian* varieties R498 and IR64^29,43^. The databases included rice gene expression data derived from RNA sequencing (RNA-Seq) analyses of different tissues, such as aleurone, anther, callus, leaf, panicle, pistil, root, seed, and shoot, at different growth stages, as well as under a variety of biotic and abiotic treatments (such as Cd, Pi, drought, cold and salt). The expression profiles of all rice CAX genes are available in the Table S5. The gene expression levels were calculated based on the log transformation of the FPKM values. Heatmaps of the expression patterns of all rice CAX genes were plotted using TBtools.

### Construction of gene gcHap networks of rice CAX genes

When compared in IRGSP-1.0, all SNPs identified within the CDS regions of the six OsCAX genes were used to obtain the number of gene CDS haplotypes (gcHapN) for each of the OsCAX genes. Shannon’s equitability (*E*_*H*_) was used to evaluate the level of gcHap diversity at all rice CAX loci in specific rice populations or the whole species^44^. A haplotype network was built for each of the rice CAX genes based on pairwise differences between two adjacent gcHaps using the R package “pegas”^45^. To plot the network, we used only the major haplotypes, each carried by at least 50 accessions in the 3KRGP accessions. The network was produced using statistical parsimony, such that the most closely related haplotypes were connected first via the smallest number of mutations^46^.

To understand how modern breeding in past decades affected the gcHap diversity of rice CAX genes, we compared the differences in *E*_*H*_ estimates and frequencies of the predominant gcHaps between 732 *Xian* (*indica*) landraces (LANs-*Xian*) and 328 modern *Xian* varieties (MVs-*Xian*) and between 358 *Geng* (*japonica*) landraces (LANs-*Geng*), and 139 modern *Geng* varieties (MVs-*Geng*) (https://www.rmbreeding.cn/Index)^47^ using an R script (https://github.com/isaac-Tsang/gcHap_diversity_LANs_MVs-) and Z tests^27,48^. We also compared the differences between LANs-*Xian* and MVs-*Xian* and between LANs-*Geng* and MVs-*Geng* for possible newly emergent gcHaps at each of the CAX genes in rice MV varieties. Finally, the data above were plotted using GraphPad Prism 9 software and the “ggplot2” package in R.

### Genome-wide association study (GWAS) based on SNPs/gchaps

Based on the haplotype network analysis, the predominant gcHaps of the CAX genes in 3KRGP were determined and analyzed for their associations with several agronomic traits, including culm length (CL, cm); culm number (CN, count); grain length (GL, mm); 1000-grain weight (TGW, g); grain width (GW, mm); and days to heading (HD, day) from the IRRI (snp-seek.irri.org/)^49^ and RFGB databases (https://www.rmbreeding.cn/)^47^. The major haplotypes of all rice CAX genes, each carried by at least 50 accessions in the 3KRGP accessions, were obtained using an R script (https://github.com/isaac-Tsang/haplotype_network). Finally, the associations of major haplotypes with these agronomic traits across 3010 diverse rice accessions were achieved using an R script (https://github.com/isaac-Tsang/functional-importance-). The differences between the haplotypes were calculated by a two-way analysis of variance (ANOVA), and statistically significant differences were based on Duncan’s multiple range test at *P* < 0.05. GraphPad Prism 9 software was used to generate the phenotype and haplotype box graphs from the GWAS analyses.

GWAS was conducted to determine the associations of the major gcHaps for each of the CAX genes conferring salt tolerance, using historical phenotypic data for salt tolerance at the germination stage of the 478 rice accessions in 3KRG^50^. Moreover, validation of the associations of the CAX genes with salt tolerance was conducted using the agronomic trait data of 496 Huanghuazhan (HHZ) ILs under salt tolerance. The GWAS results are available in the Table S6. Manhattan plots of the GWAS results were analyzed using a mixed linear model (MLM) and plotted using the R package “rMVP”. We defined the LD decay distance to be chosen in such a way that the LD coefficient (r^2^) is reduced to half of its maximum value^51^. The significant SNP was defined as the SNP with the *P* value < 10^–5^. The candidate gene was identified within 200 kb upstream and downstream of the significant SNP.

### Genome-wide variant detection and analysis of SNPs, SVs, and PAVs in the rice CAX family

For the 3KRG SNP dataset, we selected the 4.8mio SNPs subset (https://snp-seek.irri.org/) with 478 rice accessions^49,50^, and 3,355,717 SNPs were filtered. For HHZ ILs, we used “gatk” to call SNPs and “delly” to call the SVs with default parameters^52^. 1,345,828 SNPs were filtered based on the following criteria: (1) minor allele frequencies (MAFs) ≥ 0.05; (2) missing rate < 20%. For 111 high-quality rice genomes, we used “delly” to call the SVs of the CAX gene family with default parameters, and the genomes of nine HHZ IL parents were included in the 111 rice genomes.

## Results

### Phylogenetic relationship of CAX genes among different species

**Figure 1:**
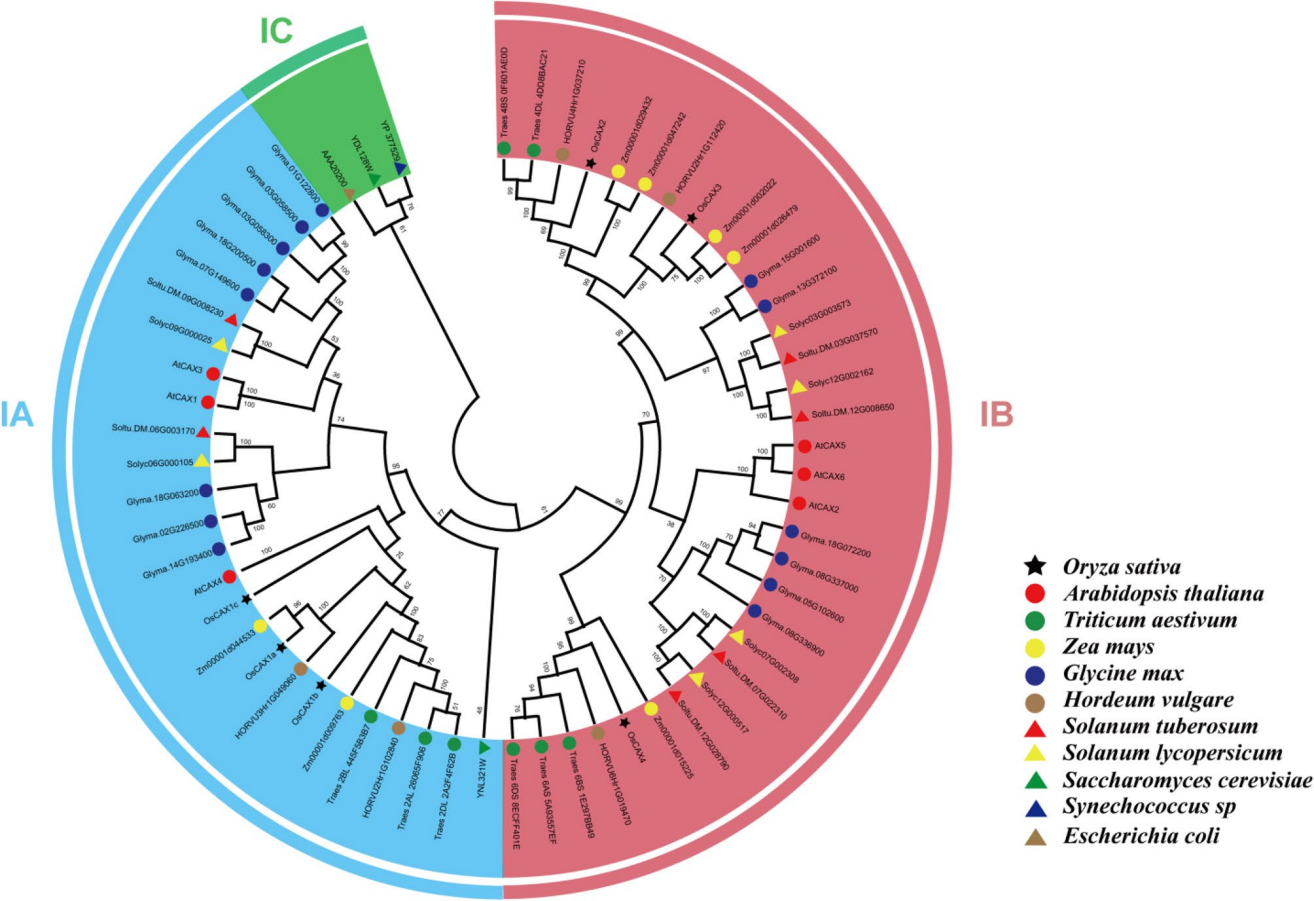
The phylogenetic relations of the CAX gene family in different species. The phylogenetic relations of CAX from *Oryza* *sativa* based on the Os-Nipponbare-Reference-IRGSP-1.0 reference genome and other species, including *Arabidopsis* *thaliana* (At), *Triticum* *aestivum* (Ta), *Zea* *mays* (Zm), *Glycine* *max* (Glyma), *Hordeum* *vulgare* (Hv), *Solanum* *tuberosum* (St), *Solanum* *lycopersicum* (Sl), *Saccharomyces* *cerevisiae* (Sc), *Synechococcus* sp (Ssp), and *Escherichia* *coli* (Ec). The 11 species are depicted by different colored shapes.

To investigate the evolutionary relationships of OsCAXs with CAXs from different species, an unrooted phylogenetic tree was constructed based on the aligned amino acid sequences of 62 CAX proteins (Fig. 1). The 62 CAX proteins were classified into three large clusters (IA, IB, and IC) containing 26 (IA), 33 (IB) and three (IC) proteins, respectively. Among the six OsCAX proteins, *OsCAX2*, *OsCAX3*, and *OsCAX4* were grouped in three subgroups of cluster IB. *OsCAX2* and *OsCAX4* showed high homology with *HORVU4Hr1G037210* and *HORVU6Hr1G019470* of *Hordeum vulgare* in Cluster IB. *OsCAX1a*, *OsCAX1b*, and *OsCAX1c* were classified in the same subgroup of Cluster IA, though the former two were more closely related to each other. Clusters IA and IB contained two and five maize CAX genes related to *OsCAX1a*-*1c*, and *OsCAX2*-*4*, respectively. Moreover, *OsCAX1a* and *OsCAX1b* displayed high homology with *HORVU3Hr1G049060* in Cluster IA. The soybean CAX family comprised 14 genes. And the homology analysis of CAX in bacteria and yeast was consistent with previous studies, indicating a separate third type. *YNL321W* in yeast may be more closely related to plants. The prediction of protein structures in rice, bacteria, and yeast revealed that *OsCAX1a*, *OsCAX1b*, and *OsCAX1c*, *OsCAX2*, and *OsCAX3* have similar protein structures, each with 11 transmembrane (TM) helices, while *OsCAX4* has only nine helices (Fig. S1). However, there were significant differences between the protein structures of CAX in rice and bacteria and yeast. CAXs in non-plant species were more tightly packed in membranes than those in rice, suggesting functional differentiation in the CAX family.

When the genomic locations of the six rice CAX genes were examined, we found that two pairs of homologous OsCAX genes, *OsCAX1a/OsCAX1b* and *OsCAX2/OsCAX3*, were located on segments of chromosomes 1/5 and 3/4 (Fig. S2), consistent with the duplicated chromosomal segments from the whole genome duplication (WGD) event in Poaceae inferred to have occurred about 65 million years ago (mya)^53^. The locations of *OsCAX1a* and *OsCAX1b* on rice chromosomes 1 and 5 suggested that additional duplication events occurred during the diploidization process after WGD. Moreover, the Ka/Ks ratios of all rice CAX pairs were <  < 1.0, suggesting that all rice CAX genes had gone through strong purification selection during evolution (Table S7). These results suggest that the formation of the six rice CAX genes resulted from the well-known WGD event in Poaceae, while the close relationships of the rice CAX genes with maize CAX genes suggest that the rice CAX genes might share similar functionalities to those in maize. But whether there is a similar function between them still needs to be further verified by experiments.

### Genetic diversity of rice CAX genes

To reveal the total genetic diversity of the six OsCAX genes in rice populations, we first examined their PAV diversity in 453 representative accessions of 3KRGP^54^. Of the six CAX genes, *OsCAX2* and *OsCAX3* were core genes in 453 accessions without PAV variation (Table S8). *OsCAX1a* was present in 453 accessions but absent in six *Geng* accessions of the subtropical group (GJ_subtrop_). *OsCAX1b* was present in all accessions except four accessions of population ADM. Similarly, *OsCAX1c* was present in all accessions except one of the *Xian* accessions from South Asia (XI2). *OsCAX4* was present in all rice accessions except one *Xian* accession from China (XI1). We also examined their PAV diversity in the HHZ IL parental genomes, found that no PAVs were detected in all OsCAX genes (Table S9). This suggests that all six rice CAX genes are functionally important. For the correlation between geographic environmental conditions and CAXs, we analyzed the frequency of rice CAX genes existence in varieties at different geographical locations (Table S10). Remarkably, a significant geographic variation is observed in the distribution of the *OsCAX1a* and *OsCAX1b* genes. Five out of the six accessions lacking the *OsCAX1a* gene are from Southeast Asia, while the remaining one of the six accessions is from South Asia. All four accessions lacking the *OsCAX1b* gene are from Africa. The other four OsCAX genes showed almost no differences in geographical locations.

**Figure 2:**
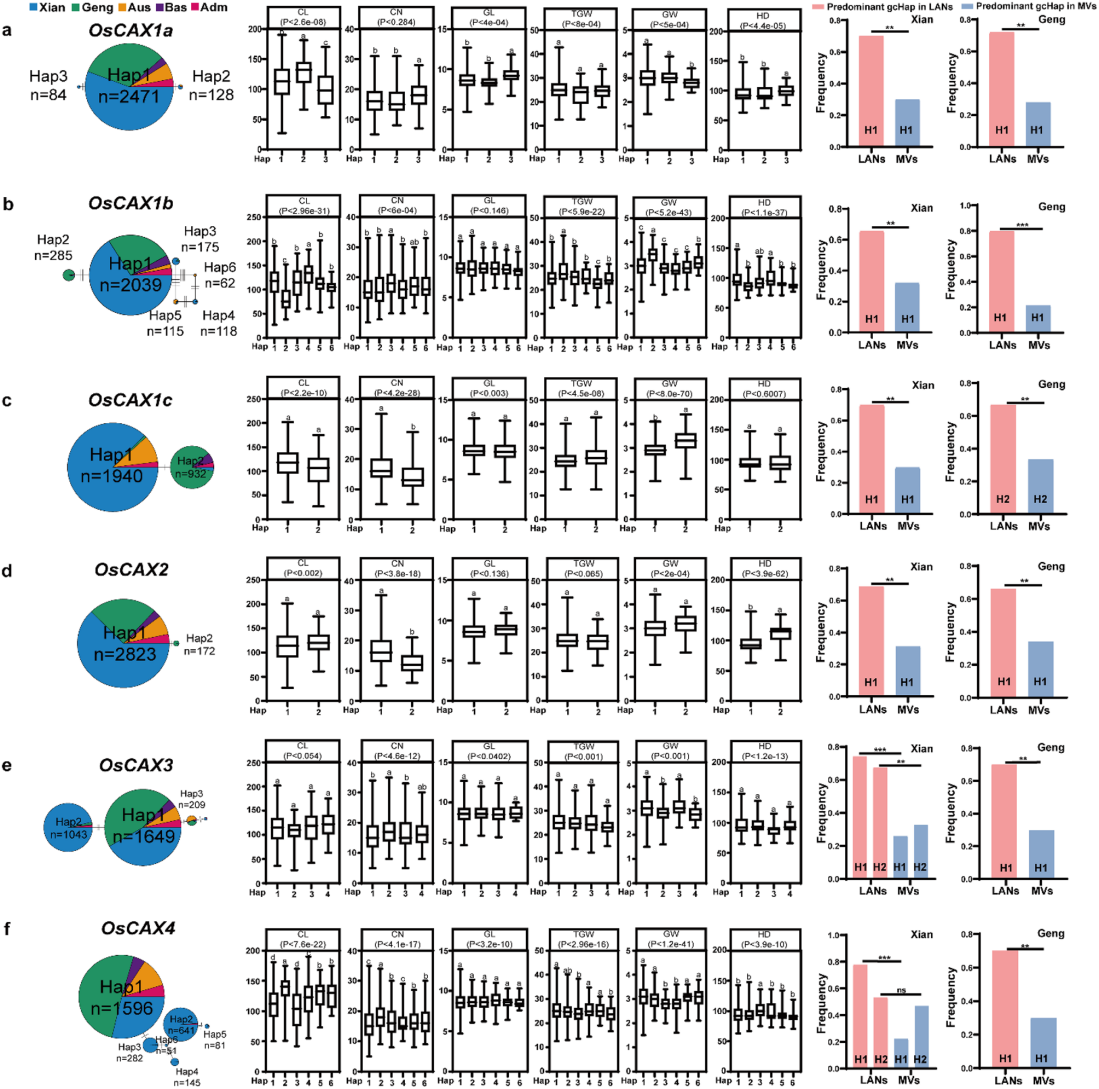
Haplotype networks of OsCAX gene family and their associations with six traits in 3KRG. (**a**) *OsCAX1a*, (**b**) *OsCAX1b*, (**c**) *OsCAX1c*, (**d**) *OsCAX2*, (**e**) *OsCAX3*, (**f**) *OsCAX4*. Within each haplotype network, two adjacent gcHaps are separated by mutational changes with hatches indicating differences between the two most related haplotypes. The right side of each gene haplotype network corresponds to the phenotypic variation among the haplotypes. Boxplots are shown for the following traits: culm length (CL, cm); culm number (CN, count); grain length (GL, mm); 1000 grain weight (TGW, g); grain width (GW, mm); and days to heading (HD, day). The *P* values under trait names indicate differences between the haplotypes assessed by two-way ANOVA, with different letters on the boxplots indicating statistically significant differences at *P* < 0.05 based on Duncan’s multiple range test. The bar charts on the right show the differences in frequency of the predominant gcHaps between landraces (LANs) and modern varieties (MVs) in Xian and Geng. Chi-square tests were used to determine significant differences in the proportions of the same gcHap between the different populations with ****P < 0.0001, **P < 0.01, *P < 0.05, and n.s., not significant.

We then analyzed the gcHap diversity of the six OsCAX genes in 3KRG (Table S11). Of the six rice CAX genes, *OsCAX1a* and *OsCAX2* were highly conserved (*E*_*H*_ = 0.012 and 0.058, and gcHapN = 8 and 20), each with a single predominant allele plus three or two major alleles in rice populations (Fig. 2). The remaining four OsCAX genes were moderately diverse, with *E*_*H*_ (gcHapN) ranging from 0.120 (41) for *OsCAX1b* to 0.197 (83) for *OsCAX3* (Table S11). Interestingly, a significant portion of the gcHaps of the six OsCAX genes were unique for specific populations, including 19.5 *Xian*-unique gcHaps and 7.3 *Geng*-unique gcHaps. *OsCAX1c* was the only *Xian*–*Geng* divergent gene having two predominant alleles, with Hap1 present only in all *Xian* accessions and Hap2 in all *Geng* accessions (Fig. 2). We also identified a significant level of gcHap diversity among the nine HHZ IL parents (Table S12). Specifically, the gcHapN detected in the nine HHZ IL parents was four in *OsCAX1a*, six in *OsCAX1b*, nine in *OsCAX1c* and *OsCAX4*, and three in *OsCAX2* and *OsCAX3*. Interestingly, all 34 alleles of the OsCAX genes identified in the HHZ IL parents were rare in the *Xian* accessions of 3KRG (Tables S12 and S13). To determine whether the OsCAX genes were of potential value in rice improvement, we compared the predominant gcHap frequencies between the landraces and modern varieties of subspecies *Xian* and *Geng* (Fig. 2). Surprisingly, the predominant gcHap (Hap1s) frequencies of all six OsCAX genes dropped significantly in the modern varieties when compared to the landraces in both subspecies, replaced by either rare ones or newly emerged ones that were absent in the landraces but present only in modern varieties. This clearly indicates that the ancient predominant alleles of all six OsCAX genes were not favored by artificial selection during the modern breeding of both subspecies.

We then analyzed the SVs in the six OsCAX genes in 453 representative accessions of 3KRGP with a sequencing depth of 20X and detected an around 4-kb deletion in *OsCAX1c* and a 300 bp deletion in *OsCAX3*. Because of the low power in detecting SVs from the NGS-generated short sequences, we analyzed 111 high-quality genomes from third-generation sequencing (TGS) technology^29^ and detected 636 SVs of various sizes in the OsCAX genes. These SVs included 48.8% deletions, 48.9% insertions, 2.1% duplications, and one copy number variation ranging from 35 to 35,370 bp. Among these SVs, 58% occurred in intergenic regions, 33% in upstream promoter regions, and 9% in genic regions. Specifically, the number of SVs detected was 461 in *OsCAX1a*, 90 in *OsCAX4*, 64 in *OsCAX3*, 15 in *OsCAX1c*, four in *OsCAX2*, and two in *OsCAX1b* (Table S14). In the HHZ IL parental genomes, SVs were detected in all OsCAX genes except *OsCAX1c* (Fig. S3 and Table S15). Multiple small insertions and deletions in the intergenic region of *OsCAX1a* were detected among all parents except for IR50 and IR64, ranging from two SVs in HHZ, OM1723, and Phalguna to ten SVs in IR64. In *OsCAX1b*, we detected a single deletion of about 810 bp in the promoter region of PsBRC28, IR64, OM1723, and Phalguna but not in the other five parents. A single deletion of about 266 bp was detected in the promoter region of *OsCAX2* in all nine parents. In *OsCAX3*, a 30 bp deletion and a 385 bp insertion were detected in the promoter regions of all parents except IR64, in addition to a 51 bp deletion in the intergenic regions of HHZ and Teqing. A single insertion of sizes ranging from 208 to 251 bp in nine parents was identified in the promoter region of *OsCAX4*. There were one or more SVs in all six OsCAX genes among the different rice accessions, particularly in *OsCAX1a*, and all of these SVs occurred in the intergenic or intronic regions.

Another aspect of the genetic diversity and functional differentiation of the six OsCAX genes was reflected by the CRE variation in the 2-kb promoter regions because the number and type of CREs in the promoter region of a gene are known to determine its expression profile, its phenotypic effects, and how it is regulated. Table 1 shows the 25 CREs predicted in the 2-kb regions of the six OsCAX genes in the 50 representative high-quality rice genomes (25 accessions for *Xian* or *Geng*). These CREs were roughly classified into five large functional categories (Fig. S4) according to their binding transcriptional factors (TFs), including six light-responsive elements, four stress-responsive elements, eight hormone-responsive elements, five developmental-related elements, and two MYB-related elements. However, considerable variation was present in the type and copy number of specific CREs in the promoters of the six OsCAX genes and their frequencies in populations *Xian* and *Geng.* Of the 25 CREs, eight CREs (Box-4, MYB, MYC, ARE, STRE, as-1, CGTCA-motif, and TGACG-motif) were common ones detected in the promoters of all six OsCAX genes, each in a significant portion of the examined rice accessions. ARBE and ERE were also common and present in six of the OsCAX genes, except *OsCAX1c* and *OsCAX1b*. Most of the common CREs belonged to the abiotic stress and hormone-responsive groups, consistent with their expected primary functions. The remaining CREs were highly specific, each present in promoters of 1–4 of the OsCAX genes in all rice accessions or a portion of a rice population, indicating that they contribute to the specific functions of different OsCAX genes. For example, a light-responsive CRE, GA-motif, was detected in *OsCAX2* in 90% of *Geng* and 50% of *Xian* accessions, while ARE was detected in *OsCAX1a* and *OsCAX1b* in approximately 50% of the rice accessions and in *OsCAX4* in 40% of *Geng* accessions. LTR was detected in *OsCAX1a*, *OsCAX2*, and *OsCAX4* in > 50% of rice accessions. We observed minor and quantitative differences between populations *Xian* and *Geng* regarding the average number and type of common CREs in the OsCAX gene promoters, indicating their evolutionary conservativeness. However, qualitative differences between the *Xian* and *Geng* accessions were observed in many specific CREs, particularly in the *OsCAX2* and *OsCAX4* promoters. In the *OsCAX4* promoter, four light-responsive CREs (AE-box, G-box, I-box, and MRE), a TGA-element, and an RY-element were present in all or most accessions of one subspecies but not in the other. Highly significant quantitative differences were also detected between the *Xian* and *Geng* accessions for the frequencies of the three hormone-responsive elements in the *OsCAX4* promoter. In the *OsCAX2* promoter, four CREs (I-box, ARE, DRE-core, and TGA-element) were present in most *Geng* accessions but absent in the *Xian* accessions. These results suggest that the emergence of *OsCAX2* and *OsCAX4* might be accompanied by the well-known *Xian*–*Geng* subspecific differentiation and might have contributed to the adaptations of *Geng* rice to long-day and colder environments. In order to explore the difference of monocots and dicots, we also analyzed the CREs between them and observed a higher average number of CRE species in monocots, particularly in development-related CREs (Fig. S4). This could indicate that these CREs were more effectively regulated by hormones in monocotyledons to facilitate their growth. Furthermore, stress-responsive CREs were less prevalent in monocots compared to dicots, indicating that despite their faster growth, monocots are more vulnerable to environmental stress.

**Table 1:**
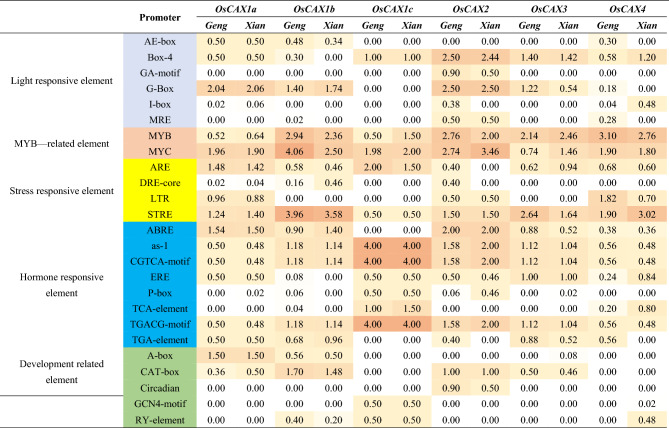
The type and frequency of different CREs in the 2-kb promoter regions of six OsCAX genes in 50 rice accessions.

In addition, we analyzed of the domains of rice, Arabidopsis, bacteria, and yeast CAX genes, which revealed that *Na_Ca_ex* is present in all genes, indicating a relation to family characteristics and response to stress through the regulation of calcium ion transport. Furthermore, we observed that *FA_desaturase* and *Glyco_hydro_59* were exclusive to plants, while *YccF*, *Na_K-ATPase*, and *SieB* were exclusive to non-plant species. This observation suggests functional differentiation of CAX genes (Fig. S5).

### Functional differentiation of rice CAX genes

**Figure 3:**
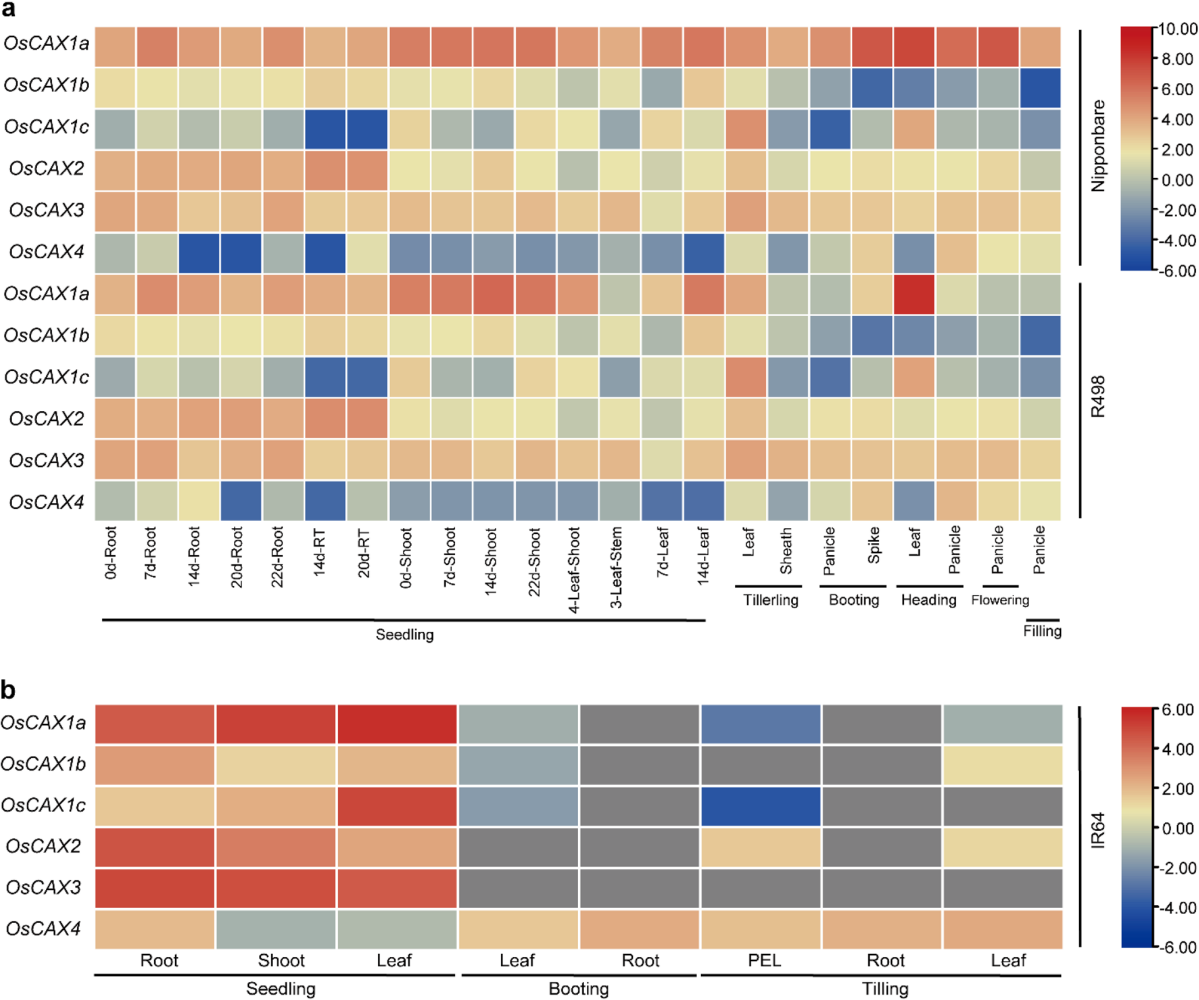
Expression profiles of the OsCAX genes in different tissues and developmental stages. (**a**) Different tissues and developmental stages expression patterns of the OsCAX gene family in Nipponbare (Geng) and R498 (Xian). (**b**) Different tissues and developmental stages expression patterns of the OsCAX gene family in IR64 (Xian).

**Figure 4:**
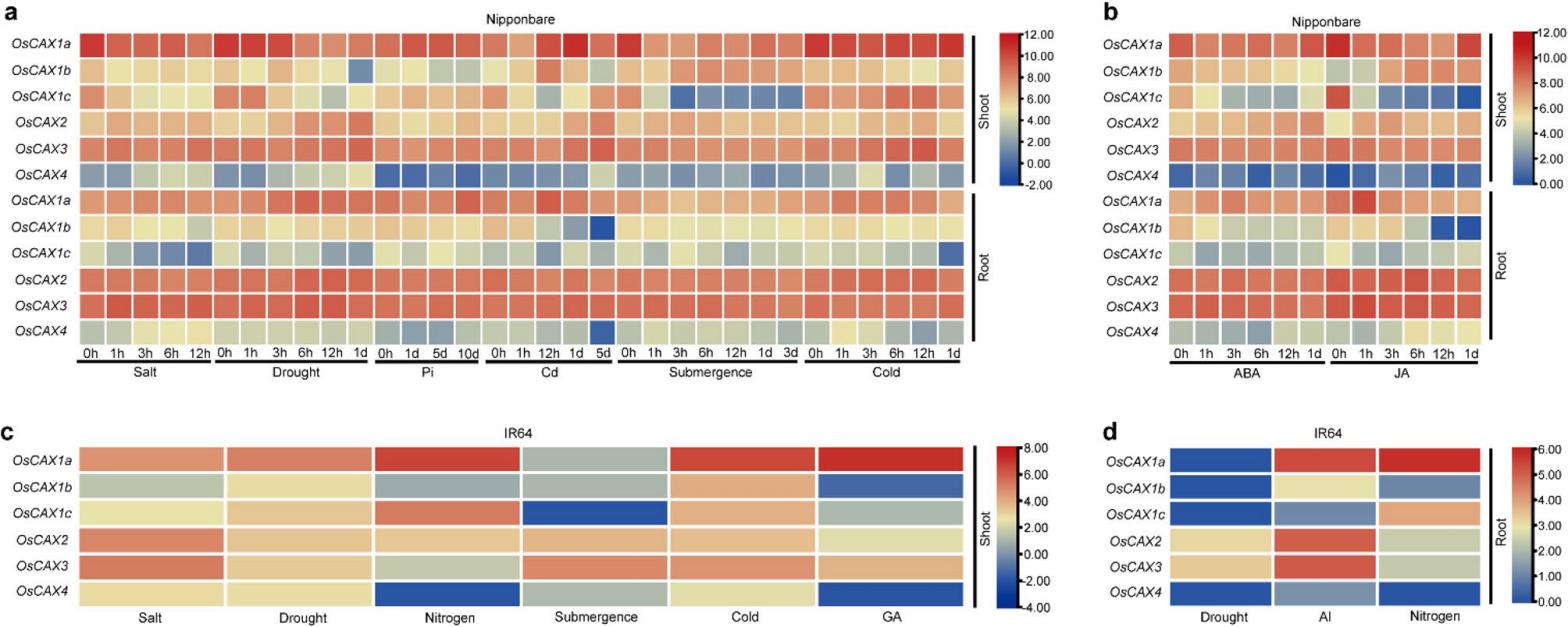
Expression profiles of the OsCAX genes under different abiotic stresses. (**a**) Expression profiles of OsCAX genes in Nipponbare (Geng) under salt, drought, Pi, Cd, Submergence and cold treatment in shoot and root. (**b**) Expression profiles of OsCAX genes in Nipponbare (Geng) under ABA and JA treatment in shoot and root. (**c**) Expression profiles of OsCAX genes in IR64 (Xian) under salt, drought, nitrogen, Submergence, cold and GA treatment in Shoot. (**d**) Expression profiles of OsCAX genes in IR64 (Xian) under drought, Al and nitrogen treatment in root.

The promoter CRE contents and gene coding sequence divergence of the six OsCAX genes from the above analyses suggest their obvious functional differentiation. To obtain additional evidence for this, we examined the expression profiles of the OsCAX genes (Figs. 3 and 4) using the following observations. In Nipponbare (*Geng*), different OsCAX genes showed strikingly different expression patterns at different developmental stages and in response to different abiotic stress or to ABA and JA treatments. *OsCAX1a* was expressed strongly and constitutively in all plant tissues at all developmental stages and under all abiotic stress or ABA/JA treatments. *OsCAX2* and *OsCAX3* showed similar expression patterns to *OsCAX1a*, although their expression levels were slightly weaker (Figs. 3 and 4), suggesting their important roles in the tolerance of rice to these abiotic stresses. In contrast, the expression of the remaining three OsCAX genes was more tissue or developmentally specific. The expression of *OsCAX1b* was strong at the seedling stage but became weaker at the reproductive stage (Fig. 3), although its expression in the shoot was strongly upregulated under all abiotic stress and ABA/JA treatments (Fig. 4). The expression of *OsCAX1c* was weak in the roots under all abiotic stress and ABA/JA treatments. However, its expression in shoots was strongly upregulated under all abiotic stress but downregulated under submergence and ABA/JA treatments (Figs. 3 and 4). The expression of *OsCAX4* was weak at the seedling stage and strong in the panicles. Its expression in shoots was also weak under all abiotic stress and hormone treatments. However, its expression in roots was strongly upregulated under salt and JA treatments, suggesting its possible role in rice salt tolerance.

The OsCAX genes in *Xian* variety R498 showed similar expression patterns as in Nipponbare except for *OsCAX1a*, whose expression was weaker at the booting and flowering stage. In IR64 (*Xian*), the six OsCAX genes showed similar expression patterns in terms of tissue and developmental specificities, except that the expression of *OsCAX1a* and *OsCAX2* were strongly upregulated under nitrogen and aluminum stress. Additionally, in IR64, the expression of all six OsCAX genes was strongly upregulated in shoots but strongly downregulated in roots under drought, except for *OsCAX2* and *OsCAX3*, whose expression in roots was also strongly upregulated under drought and GA treatments. *OsCAX4* showed minimal response to nitrogen and GA treatments (Figs. 3 and 4). These results indicated that the functional differentiation of the OsCAX genes was at least partially reflected by their different expression patterns. We analyzed the expression differences between Nipponbare and R498 at various growth stages, as well as between Nipponbare and IR64 under different stress conditions (Figs. S6 and S7). No significant expression difference was found between Nipponbare and R498 at different growth stages. However, *OsCAX1a* showed significant upregulation in the panicle of Nipponbare from the booting to filling stage. This suggests that *OsCAX1a* may be specifically expressed in the panicle, consistent with Gan et al.'s study indicating its involvement in rice panicle development^36^. Under stress treatments, the expression levels of six rice CAX genes in Nipponbare were higher than those in IR64, possibly due to the fact that IR64 is a stress-sensitive variety. Therefore, there is a connection between gene expression and the characteristics of the variety.

### Associations of OsCAX genes with agronomic traits and abiotic stress tolerance

To gain evidence of the functional importance of the OsCAX genes, we performed GWAS analyses to detect possible associations between the major alleles of the six OsCAX genes and several agronomic traits. Major alleles at *OsCAX1a* were significantly associated with CL (*P* = 4.1 × 10^−8^) (Fig. 2a). Very strong associations were detected for major alleles in *OsCAX1b* with CL (P = 3.0 × 10^−31^), TGW (*P* = 5.9 × 10^−22^), GW (*P* = 5.2 × 10^−43^), and HD (*P* = 1.1 × 10^−37^) (Fig. 2b). Major alleles in *OsCAX1c* were strongly associated with GW (*P* = 8.0 × 10^−70^) and CN (P = 4.2 × 10^−28^) but weakly associated with CL (*P* = 2.2 × 10^−10^) (Fig. 2c). For *OsCAX2*, strong associations were detected with CN (*P* = 3.8 × 10^−18^) and HD (*P* = 3.9 × 10^−62^) (Fig. 2d). *OsCAX3* was also associated with CN and HD, but the associations were weaker than *OsCAX2* (Fig. 2e). The major alleles in *OsCAX4* were significantly associated with all six traits, although the association was strongest for GW (Fig. 2f).

Using the previously reported phenotypic data of salt treatment during the germination stage in 3KRG^50^ and agronomic traits under salt treatment in HHZ ILs, we performed GWAS to determine whether the six OsCAX genes were associated with salt tolerance (Fig. S8). *OsCAX1a* was associated with agronomic traits (HD, days to heading) under salt treatment (Fig. S8c). *OsCAX1c* was strongly associated with the 48 h imbibition rate at the germination stage (Fig. S8a) and panicle length under salt stress (Fig. S8b).

## Discussion

Despite the great achievements in the global efforts of rice functional genomics over the past decades, applications of this theoretical research progress to rice improvement remain very limited for two primary reasons. First, cloned rice genes still account for a small portion of the total genes in the species. Second, the current information on most cloned genes is incomplete or even lacking regarding the number of traits affected, the phenotypic differences among major alleles of each rice gene, and in particular, the functional/genetic relationships between and among different genes (epistasis) /alleles (dominance) and their interactions with the environment. Thus, the greatest challenge or question is how to obtain the most genetic information for genes of unknown function required for improving complex traits through breeding by design (BBD) without going through the tedious process of gene cloning. In this study, we demonstrated a highly efficient strategy for obtaining important information on the functionalities and phenotypic effects of six OsCAX genes. Differing from the classical gene cloning approach, which typically follows single genetic variations of large phenotypic effects on specific traits of interest, this strategy started by selecting a gene family with potentially important but largely unknown functions and then revealed their possible functionalities by systematic analyses of their genetic diversity, expression patterns, associations with traits of agronomic importance from random and experimental populations using publicly available data, and responsiveness to artificial selection during modern breeding. This strategy took several steps to reveal the functionalities of the OsCAX genes that merit further discussion.

Of the six OsCAX genes, *OsCAX1a* (cluster IA) and *OsCAX2* (cluster IB) were inferred to be the original ones presumably from the two ancestor plant species before the WGD event of about 65 mya^55^, suggested by their extreme genetic conservativeness (as core genes with low genetic diversity) and greater functional versatility (constitutive expression across different plant tissues and upregulated by many abiotic stresses/plant hormones). The specificity of CAX depends on the diversity of amino acid sequences between different CAX, resulting in functional differences between CAX proteins. This was consistent with their known functions as cation transporter proteins^10,12^. Thus, the reported involvement of *OsCAX1a* in rice panicle degeneration may not be its primary function^36^. *OsCAX3* and *OsCAX4* were apparently derived from *OsCAX2* at early time during the long diploidization process after the WGD event while *OsCAX1b* and *OsCAX1c* were derived from *OsCAX1a* through unequal crossing events more recently. The tissue- and development-specific expression patterns of these four OsCAX genes strongly suggested their functions contributing the adaptation of rice plants to specific environmental factors, as reported involvement of *OsCAX1c*, and *OsCAX4* in rice cadmium (Cd^2+^) uptake and translocation^35^ and suggested by their associations with tolerance to specific abiotic stresses detected in our GWAS. Clearly, these later derived four OsCAX genes were differentiated from their ancestor ones and acquired new functions during evolution.

CAX represent a significant class of transmembrane transporters. They play crucial roles in maintaining Ca^2+^ homeostasis, enhancing abiotic stress resistance, and facilitating the transport of heavy metal ions in plants^19,56,57^. The primary substrate of CAXs is Ca^2+^, regulating cytoplasmic Ca^2+^ levels by modulating its flux across the membrane^19,58^. This involves the transportation of Ca^2+^. Reports suggest that CAXs in the tonoplast exhibit high capacity and low affinity for Ca^2+^ ^59^, indicating a potential role in scenarios with elevated cytoplasmic Ca^2+^ concentrations. *AtCAX1* is localized in the tonoplast and primarily regulates cytoplasmic Ca^2+^ concentration, exhibiting high transport activity with low affinity for Ca^2+^ ^60,61^. This indicates that *AtCAX1* may possess Ca^2+^ tolerance and facilitate Ca^2+^ uptake and transport under conditions of high Ca^2+^. Although *AtCAX2* exhibits lower affinity for Ca^2+^ compared to that of *AtCAX1*, it has a broader range of cation selectivity^60,62^. This disparity in affinity is likely attributed to variations in key amino acids involved in Ca^2+^ transport. Notably, a reported mutation from valine to leucine at position of 203 in *MdCAX3L-1* significantly enhances Ca^2+^ transport^16,63^, indicating that this position plays a pivotal role in determining its Ca^2+^ transport capacity. The variations in Ca^2+^ transport capacity correlate with the differentiation of CAXs functions, observed in both *Arabidopsis thaliana* and *Malus domestica*^60–63^. This suggests a genetic basis for this phenotypic trait. Recent studies have elucidated the molecular mechanism of CAXs in Ca^2+^ efflux and the regulation of Ca^2+^ homeostasis, which is vital for plant growth and immune responses. CAXs mediate the scavenging of excess cytoplasmic Ca^2+^ across various physiological conditions in plants^64^. Under normal soil growth conditions, plants utilize the Ca^2+^-CBL-CIPK-CAX1/3 pathway to regionalize Ca^2+^ into the tonoplast, thereby maintaining [Ca^2+^]cyt homeostasis. During immune responses, the FLS2-BAK1-BIK1/PBL1 pathway analogously activates CAX1/3, thereby modulating calcium signaling and immune responses^64^. This study focused on analyzing the physicochemical properties, structural features, phylogenetic relationships, cis-acting elements, and expression patterns of *CAXs* family members. Future research is necessary to further elucidate the specific functions of CAXs in ionic response, signal transduction, and abiotic stress tolerance, among others.

A second aspect of the OsCAX genes revealed in this study was the rich genetic diversity, particularly the rich gcHap diversity, at the four later derived ones among different rice accessions, even though the observed genetic diversity at the four OsCAX genes was lower than the average genetic diversity of all rice genes^27^. This was consistent with their expected function in the upstream regulatory role in signal transduction, and with their low gene PAVs observed in rice populations. Interestingly, we observed the significant frequency shifts of the predominant gcHaps at all six OsCAX gene loci in response to the artificial selection during modern rice breeding, suggesting that the OsCAX gene loci may also have functions involved in rice productivity, as suggested by their associations with agronomic traits detected in the GWAS. However, it remains unclear regarding which of the allele(s) were replacing the predominant one at each of the OsCAX gene loci and what specific trait(s) are associated with them. Our observations indicate that the CAX genes across various species exhibit N-terminal self-repression regulation, as extensively documented in previous reports^61,65–68^. Based on the 3D structure and Ca^2+^ transport mechanism of *ScVCX1*^69,70^, we hypothesize that the inhibitory effect exerted by the N-terminal of the CAX protein arises from its interactions with amino acids situated at specific positions within the transmembrane region. Upon alteration of key amino acids within the transmembrane region, the interaction is significantly impacted or disrupted, subsequently abolishing the inhibitory effect of the self-inhibitory region^17,61,68,71^. This hypothesis aligns with findings from other species. Deletion or disruption of the N-terminal autoinhibitory region restores the Ca^2+^ transport capacity of plant CAX proteins. SOS2 activates the Ca^2+^ transport function of CAX proteins through interactions with the N-terminal autoinhibitory region^72,73^. Similarly, the preservation of Ca^2+^ transport activity and protein structure in *ScVCX1* relies on specific amino acid interactions within transmembrane regions^69^. Recent research has demonstrated that in Arabidopsis, *AtCAX1* interacts with and is activated by *AtSOS2*, a crucial component in salt stress resistance in Arabidopsis^72^. The significance of this observation lies in the N-terminal residue of the CAX protein, as numerous proteins engage with CAX through binding to its N-terminal^74,75^.

The current knowledge indicates that the number and types of CREs in gene promoter regions represent an important aspect of gene expression regulation and thus gene functionality. Indeed, we detected a total of 25 CREs of five major functional groups, most of which belonged to the abiotic stress and hormone-responsive groups, consistent with their expected primary functions. The presence of multiple CREs in each of the OsCAX gene promoters (mean = 14.5) was a sign of their functional versatility, consistent with their key roles in the upstream of plant signal transduction. In this respect, the significant level of genetic variation in both number and type of CREs in the OsCAX gene promoters observed among the 50 high quality representative rice genomes represent another dimension of genomic diversity within and among populations which has not been well characterized for almost all rice genes. This type of genetic variation in gene promoter regions may have important phenotypic consequences, as reported in the cases of *ARE1*, *GWT2* and *GSE5* where a single deletion/insertion or a SNP mutation in the gene promoter regions caused reduced gene expression associated with large phenotypic effects on nitrogen use efficiency/grain yield (*ARE1*), grain length/yield (*GSE5*) or grain width and weight (*GWT2*)^76–78^. In this respect, three interesting observations were noted regarding the CRE number and type variation in the OsCAX gene promoter regions. First, we found that the frequencies of specific CREs present in the OsCAX gene promoters varied considerably, with eight common CREs (Box-4, G-box, MYB, MYC, ABRE, as-1, CGTCA-motif and TGACG-motif) that were present in high frequency in all six OsCAX gene promoters. For example, of the 25 CREs detected, the average copy numbers in the six OsCAX gene promoters were the highest for MYC (4.41), MYB (3.95) and STRE (3.90) across the six OsCAX genes, followed by for CGTCA-motif (3.0), as-1 (3.0) and TGACG-motif (3.0), indicating the primary functions of most OsCAX genes are involved in abiotic stresses and responsive to plant hormones. Clearly, these common CREs provide key information on which TEs involved in the regulation of these common OsCAX genes and their primary functions. Second, each OsCAX gene promoter contains a unique set of CREs in most rice accessions, which were expected to be a major contributor to its functional differentiation. For example, the *OsCAX1b* promoter contains three strong CREs (MYB, MYC, STRE), six moderately strong CREs (G-box, ABRE, as-1, CGTCA-motif, TGACG-motif, TGA-element and CAT-box). The *OsCAX1c* promoter contained five strong CREs (as-1, CGTCA-motif, TGACG-motif, ARE and MYC) and three moderately strong CREs (box-4, MYB and TCA-element). *OsCAX3* promoter contains two strong CREs (MYB and STRE), nine moderately strong CREs (Box-4, G-box, MYC, ARE, ABRE, as-1, CGTCA-motif, ERE, TGACG-motif and TGA-element). The *OsCAX4* promoter contains three strong CREs (MYB, MYC and STRE), six moderately strong CREs (Box-4, ARE, as-1, LTR, CGTCA-motif and TGACG-motif). Thirdly, the remaining CREs each had a single copy in the OsCAX gene promoters in all rice accessions or most of a specific rice population, indicating they contributing to the specific functions of different OsCAX genes. For example, a light responsive motif, AE-box is present only in the promoters of *OsCAX1a* and *OsCAX1b* of most rice accessions, but not in other OsCAX genes. A light responsive element, MRE, and the circadian element are present only in the promoter of *OsCAX2* of all rice accessions but not in other five OsCAX genes. Fourth, only minor and quantitative differences existed between populations *Xian* and *Geng* regarding the average number and type of strong and moderately CREs in the OsCAX gene promoters, indicating their evolutionary conserveness. However, qualitative differences between *Xian* and *Geng* were observed in many low copy CREs, particularly in the *OsCAX2* and *OsCAX4* promoters. In these cases, the CREs were present only in accessions of one subspecies but not in the other. In the *OsCAX4* promoter, these CREs included AE-box, G-box, MRE and TGA-element which were present in all or most *Geng* accessions not but in all or most *Xian* accessions, and the opposite was true for Box-4, I-box, ERE, and RY-element in all or most *Xian* accessions but not in all or most *Geng* accessions. Other CREs included box-4 in *OsCAX1b*, I-box, ARE, DRE-core and TGA-element of *OsCAX2* which were present in most *Geng* accessions but absent in the *Xian* accessions. These results suggested that the presence of these *Geng*-unique CREs in the *OsCAX2* and *OsCAX4* promoters might have contributed the adaptations of *Geng* rice to the long-day and colder environments. All these led us to a conclusion that the functional differentiation of different members of a gene family was at least partially achieved by recruiting new CREs in their promoter regions. This also implies that an improved efficiency of knock-in mutation by the CRISPR-case 9 technology can be achieved by creating new CREs in gene promoter regions, particularly for those conserved regulatory genes such as CAX genes. This later point should serve as a new direction in gene-editing efforts to be actively tested experimentally. Thus, future population genomic efforts should be taken to assess the types and average number of CREs in promoters of all rice genes across representative rice genomes, even though accurate assessment of gene promoter diversity within and among different rice populations requires high quality genomic sequence data.

Taking together, combined evidence from our analyses indicated that *OsCAX1a* and *OsCAX2* appear to function as general signal transporters in many processes of rice growth and development and in rice responses to diverse environments, but they might be of less value in rice improvement because of their low genetic diversity. In contrast, *OsCAX1b*, *OsCAX1c*, *OsCAX3* and *OsCAX4* each was expected to be of potential value in rice improvement because of their associations with specific rice traits, their responsiveness to specific abiotic stresses or phytohormones, and relatively high gcHap and CRE diversity. However, additional efforts have to be taken to characterize and verify the ‘desirable’ allele(s) and their phenotypic effects at each of these OsCAX genes, which can be easily achieved by the CRISPR-9 mediated knockout or knock-in mutagenesis experiments^48^.

Finally, we have demonstrated a highly efficient strategy to obtain important genetic information on genes/alleles of specific gene family through comprehensive analyses using the public genomic and phenotypic data. Obviously, the obtained information on the function and phenotypic effects of different members of the gene family is incomplete. This is virtually true for all rice genes. Fortunately, as more and more high-quality population genomic and phenotypic data of rice are accumulating in the public domain and more powerful analytic tools become available, this strategy can be used to systematically characterize all the 12,000+ rice gene families, generating tremendous amounts of genetic information more applicable to rice improvement. Also, all rice traits of agronomic importance are not controlled by single genes, but by multiple genes acting in complex signaling pathways. Thus, this strategy can be easily extended to pathway-based multi-loci analyses, focusing on all regulatory genes involved in each of important signaling pathways. One important result expected from this type of pathway-based analyses would be the more accurate prediction and quantification of the epistatic relationships between and among different genes acting in each of the signaling pathways, which will eventually allow more accurate prediction of complex trait phenotypes of breeding progenies based on genetic variation of segregating loci, an essential step to realize BBD in future.

## Conclusions

Tremendous functional differentiation of six rice CAX genes were revealed through comprehensive transcriptomic and genetic diversity analyses using population genomic and phenotypic variation data. *OsCAX2* and *OsCAX1a* were inferred to be ancient ones and function as general signal transporters in many processes of rice growth and development and in rice responses to diverse environments, while *OsCAX1b*, *OsCAX1c*, *OsCAX3* and *OsCAX4* were derived ones and each acquired new functions for specific rice traits and/or adaptation to specific abiotic stresses during evolution.

### Supplementary Information

Supplementary Information 1.

Supplementary Information 2.

Supplementary Information 3.

Supplementary Information 4.

Supplementary Information 5.

Supplementary Information 6.

Supplementary Information 7.

Supplementary Information 8.

Supplementary Information 9.

Supplementary Information 10.

Supplementary Information 11.

Supplementary Information 12.

Supplementary Information 13.

Supplementary Information 14.

Supplementary Information 15.

Supplementary Information 16.

Supplementary Information 17.

## Data Availability

Data generated and analyzed during the current study are included in this article and its supplementary information files.
