# Supplementary material for: Functional differentiation and genetic diversity of rice cation exchanger (CAX) genes and their potential use in rice improvement
Source: Sci Rep. 2024 Apr 15;14:8642. doi: 10.1038/s41598-024-58224-2 (PMC11018787; doi:10.1038/s41598-024-58224-2)
Supplement: Supplementary file 1 — Supplementary Information 1. [file 41598_2024_58224_MOESM1_ESM.docx]

**Figure**

Figure 1. The phylogenetic relations of the CAX gene family in different species.

Figure 2. Haplotype networks of OsCAX gene family and their associations with six traits in 3KRG.

Figure 3. Expression profiles of the OsCAX genes in different tissues and developmental stages.

Figure 4. Expression profiles of the OsCAX genes under different abiotic stresses.

**Table**

Table 1. The type and frequency of different CREs in the 2-kb promoter regions of six OsCAX genes in 50 rice accessions.

**Figure S**

Figure S1. Prediction of rice, bacteria and yeast CAX gene protein structure.

Figure S2. Collinearity relationship analysis of the OsCAX gene family.

Figure S3. Comparison of the nine parents of the HHZ IL population for SVs in intron/exon/UTR regions, gcHaps, PAVs, and CNVs at six CAX genes.

Figure S4. Promoter cis-regulatory element analysis of the CAX gene family in monocots (rice and maize) and dicots (cotton and soybeans). Different color boxes represent different cis-regulatory elements. Some cis-regulatory element may overlap with others.

Figure S5. Domain analysis of the CAX gene family in Arabidopsis, bacteria and yeast. Different color boxes represent different domain elements.

Figure S6. Comparison of expression profiles of the OsCAX genes in different tissues and developmental stages in Nipponbare (*Geng*) and R498 (*Xian).*

Figure S7. Comparison of expression profiles of the OsCAX genes in different stress in Nipponbare (*Geng*) and R498 (*Xian).*

Figure S8. GWAS of loci associated with germination stage and agronomic traits under salt tolerance in 3KRG and HHZ ILs, respectively.

**Table S**

Table S1. The protein sequences of CAX gene family in different species.

Table S2. Domain data of CAX genes in rice, Arabidopsis, bacteria and yeast.

Table S3. Statistics of promoter cis-regulatory elements of the CAX gene family in rice (Nipponbare), maize, cotton, and soybeans.

Table S4. 50 high-quality accessions information.

Table S5. Expression profiles of the OsCAX gene family.

Table S6. GWAS results of the association of the six rice CAX genes with salt tolerance in germination stage in the subsample of 3KRG and HHZ ILs.

Table S7. Homologous gene pair Gene 1 and Gene 2, Ka, Ks, and Ka/Ks-value of the OsCAX gene family.

Table S8. The PAVs at six rice CAX genes in different rice populations of 3KRGP.

Table S9. The PAVs at six CAX genes in the nine parents of the HHZ ILs.

Table S10. The number of *OsCAX1a* and *OsCAX1b* absence or presence accessions in different region.

Table S11. The gcHap number and Shannon’s equitability (EH) of CAX rice genes in 3KRG.

Table S12. The gcHaps at OsCAXs in the nine HHZ IL parents.

Table S13. The gcHaps at OsCAXs in the 3KRGP.

Table S14. SVs at OsCAXs detected in the 111 high-quality genomes.

Table S15. SVs at OsCAXs detected in the nine parents of HHZ ILs.
