# Supplementary material for: Functional differentiation and genetic diversity of rice cation exchanger (CAX) genes and their potential use in rice improvement
Source: Sci Rep. 2024 Apr 15;14:8642. doi: 10.1038/s41598-024-58224-2 (PMC11018787; doi:10.1038/s41598-024-58224-2)
Supplement: Supplementary file 2 — Supplementary Information 2. [file 41598_2024_58224_MOESM2_ESM.pdf]

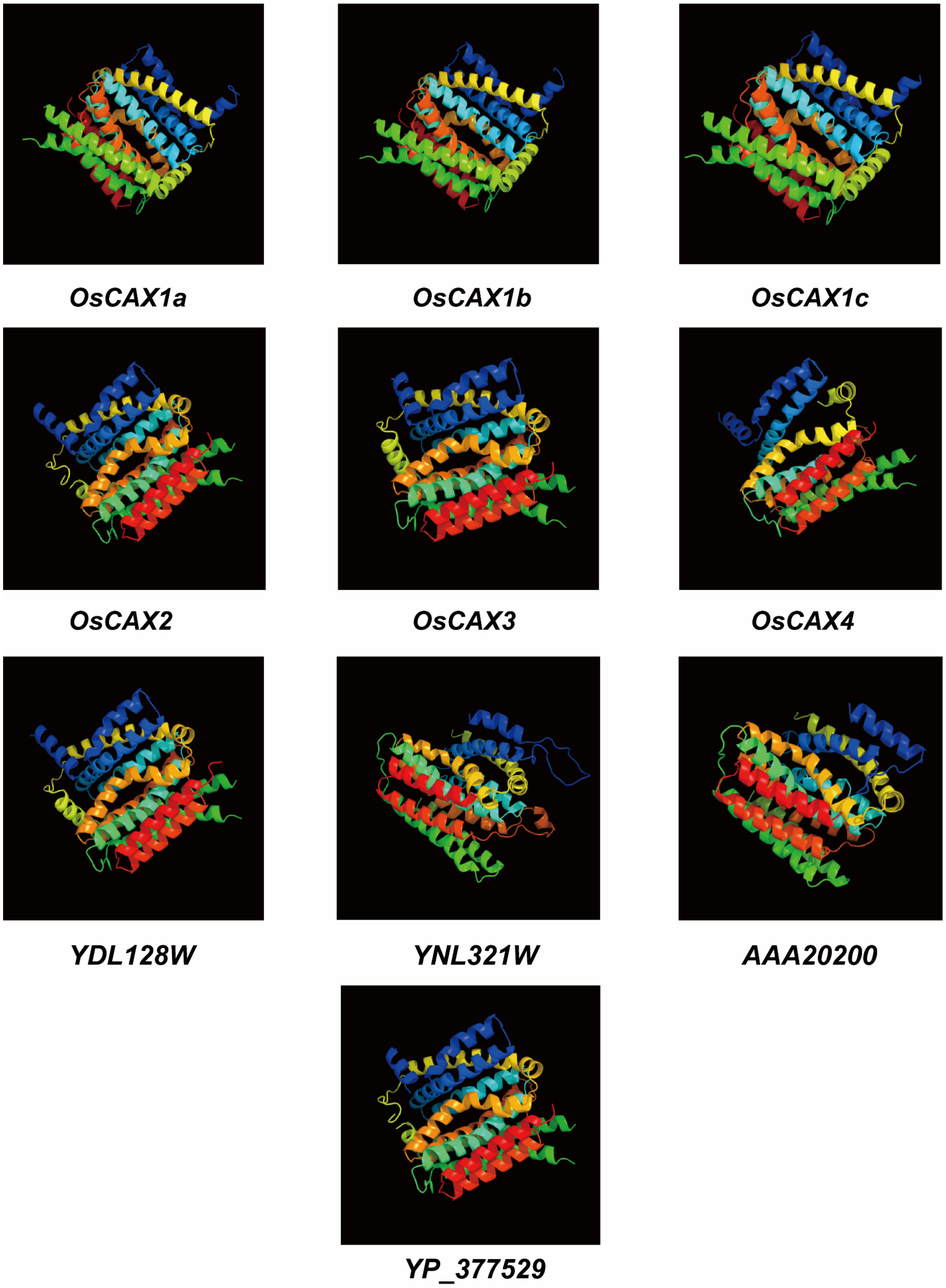

Fig. S1 Prediction of rice, bacteria and yeast CAX gene protein structure.

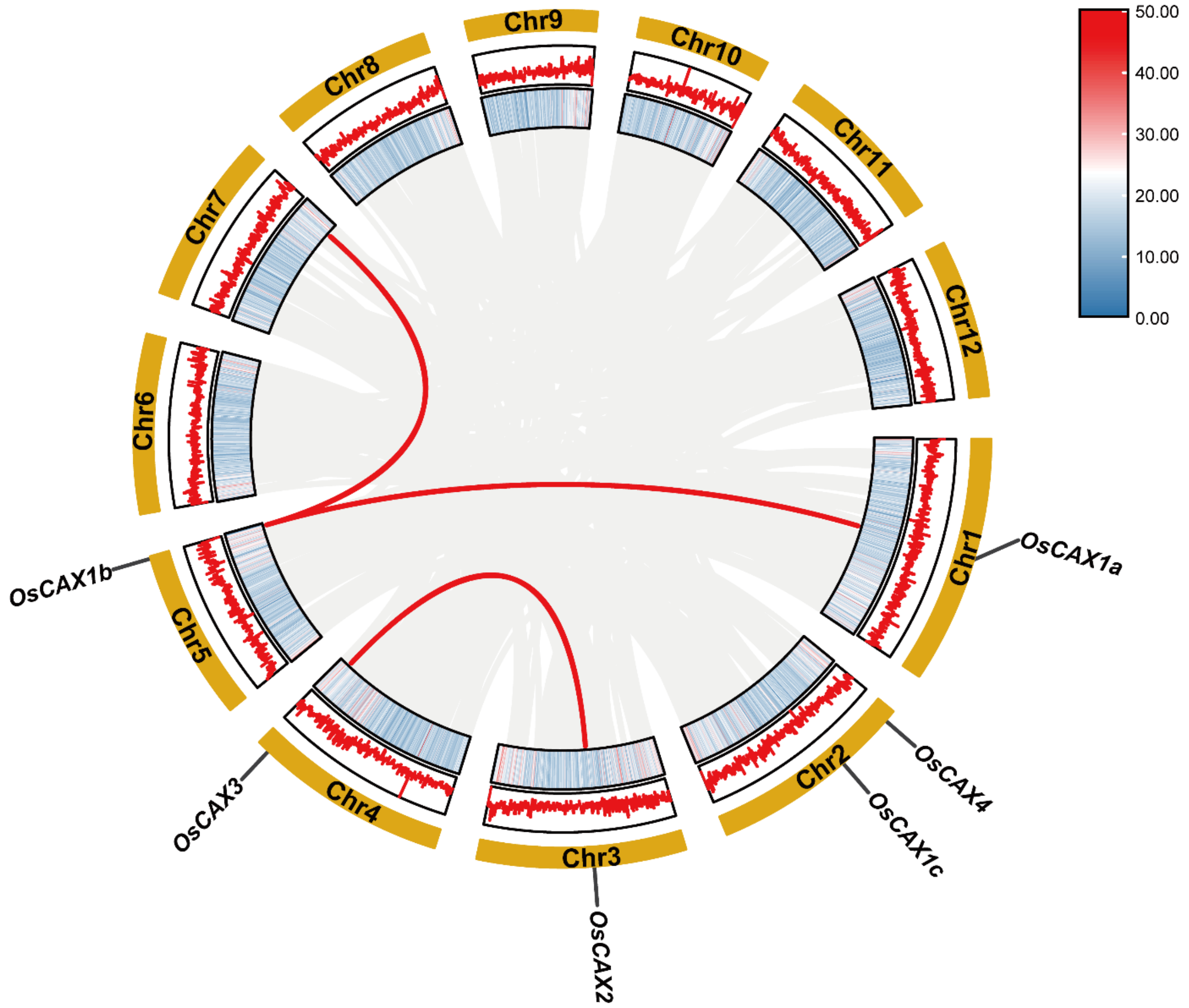

Fig. S2 Collinearity relationship analysis of the OsCAX gene family. The red lines represent the four homologous gene pairs in Oryza sativa.

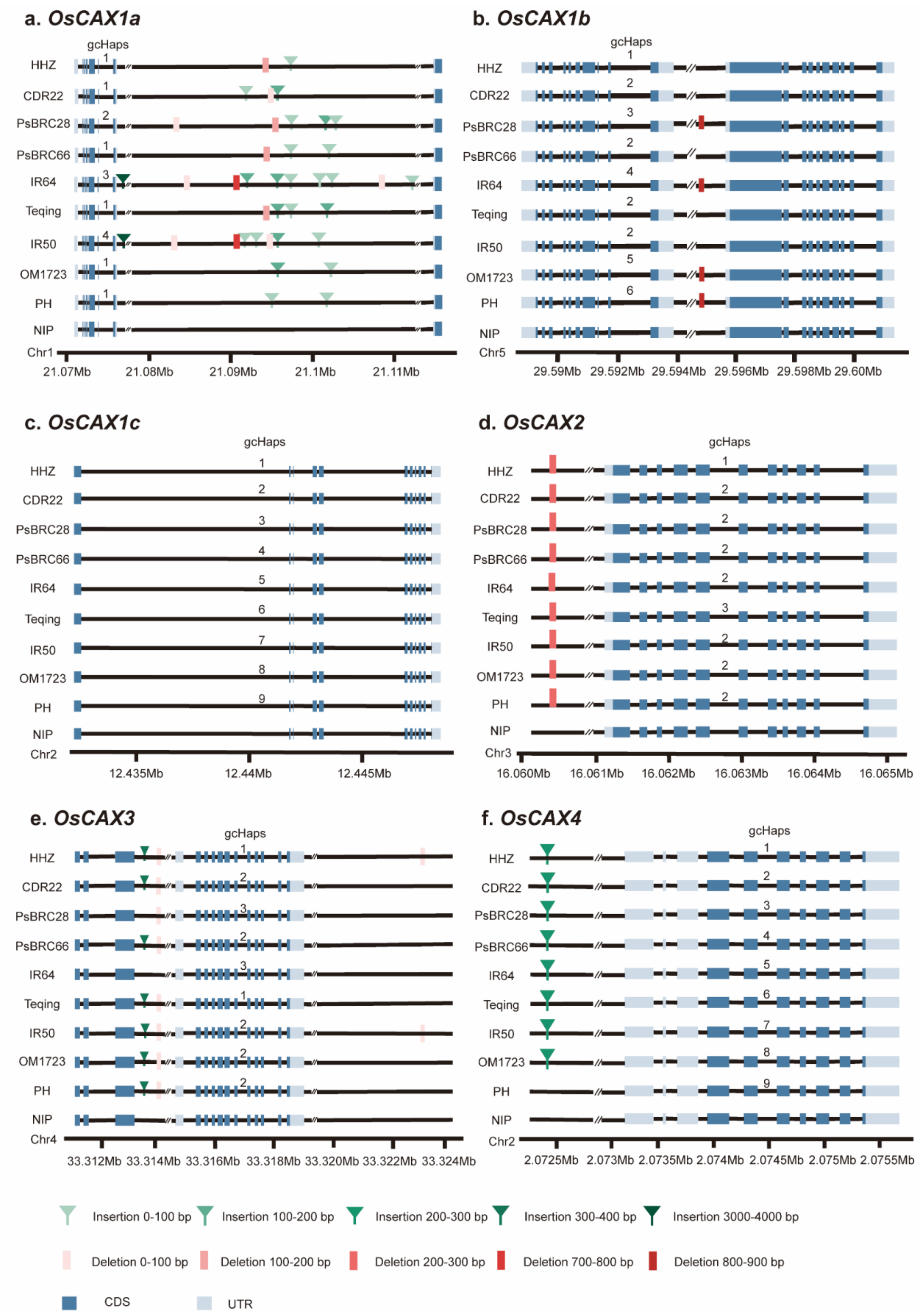

Fig. S3 Comparison of the nine parents of the HHZ introgression line population for structural variations (SVs) in intron/exon/UTR regions, gene CDS haplotypes (gcHaps), presence/absence variation (PAV) and copy number variation (CNV) at six CAX genes.

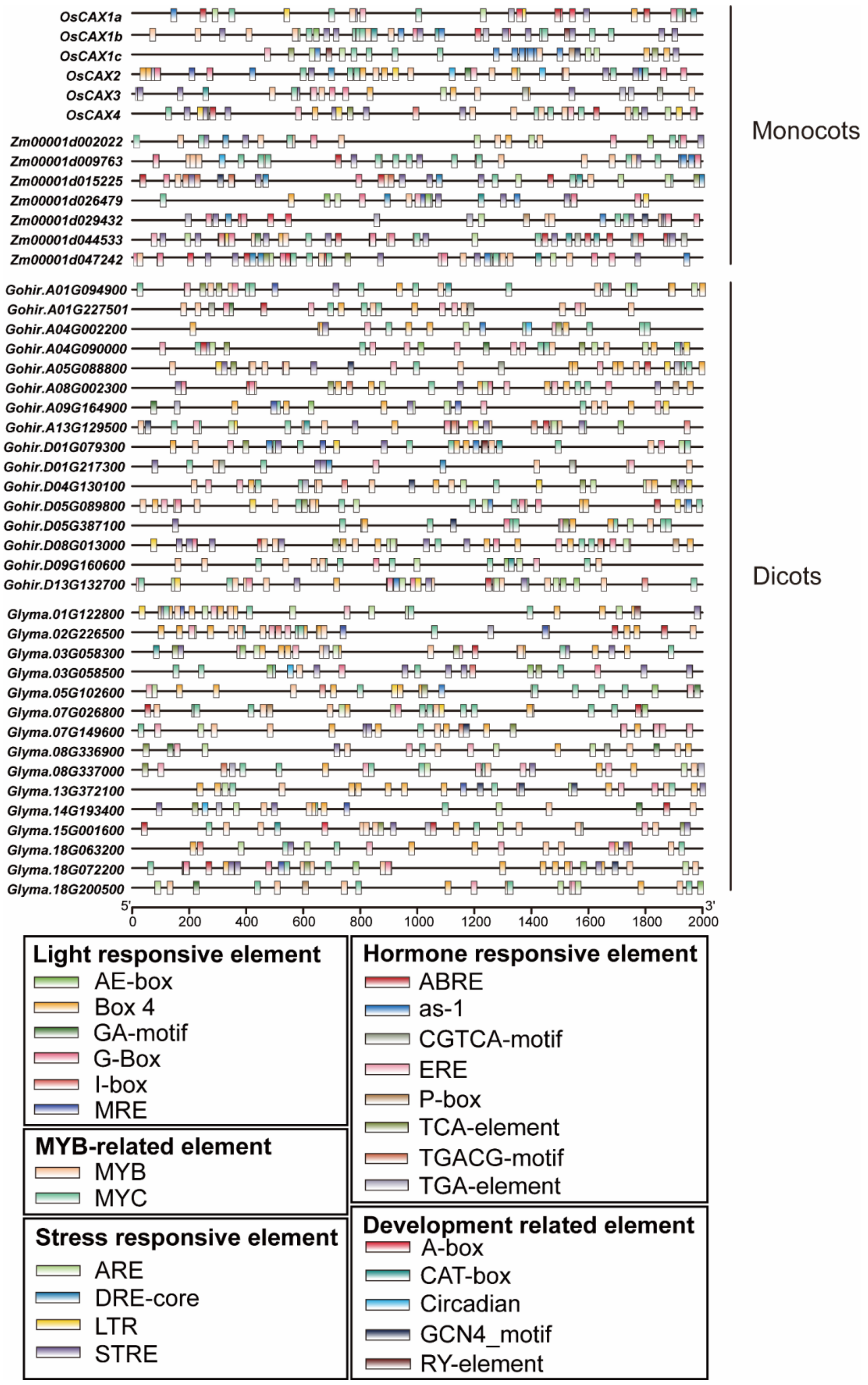

Fig. S4 Promoter cis-regulatory element analysis of the CAX gene family in monocots (rice and maize) and dicots (cotton and soybeans). Different color boxes represent different cis-regulatory elements. Some cis-regulatory element may overlap with others.

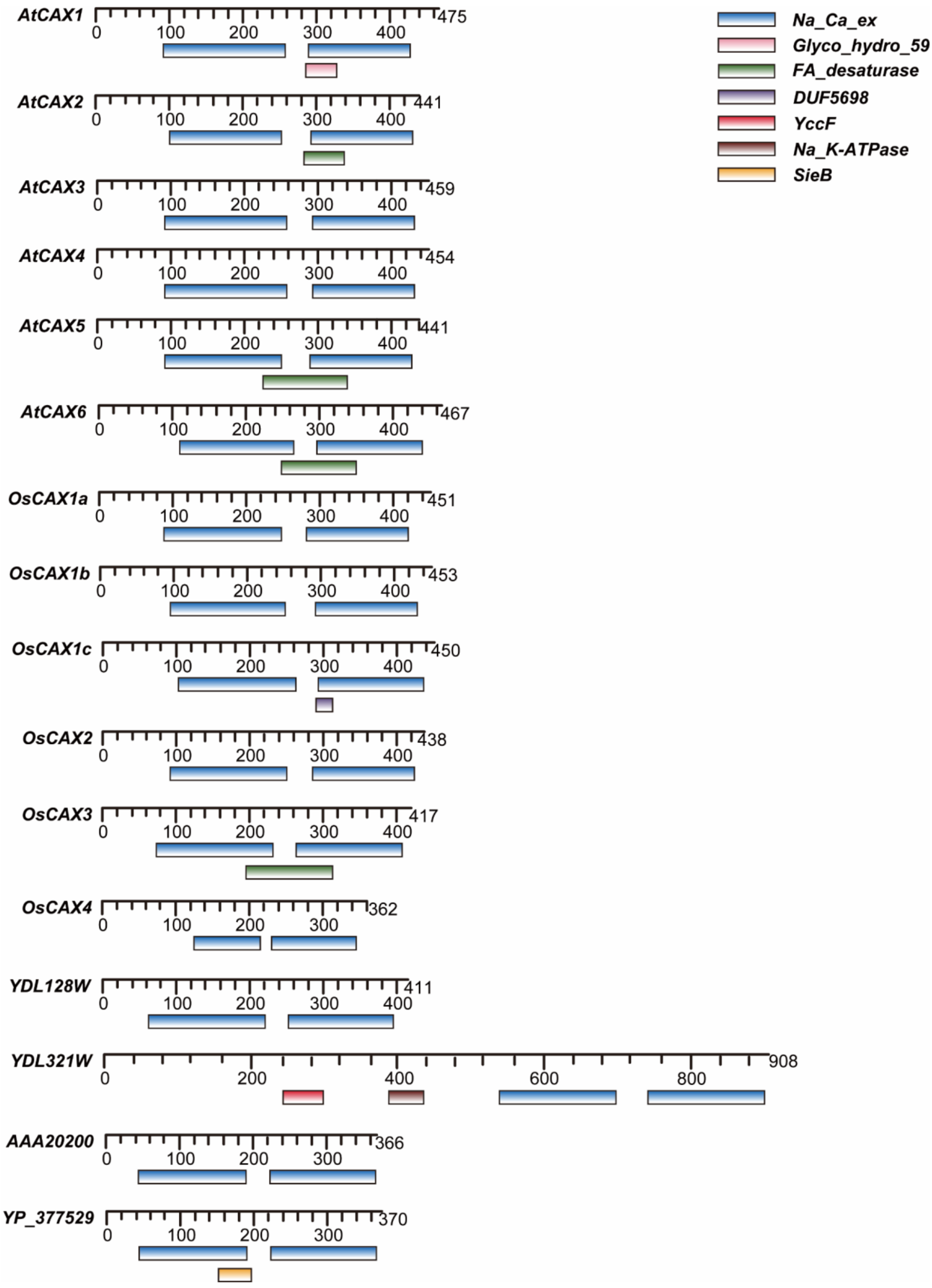

Fig. S5 Domain analysis of the CAX gene family in Arabidopsis, bacteria and yeast. Different color boxes represent different domain elements.

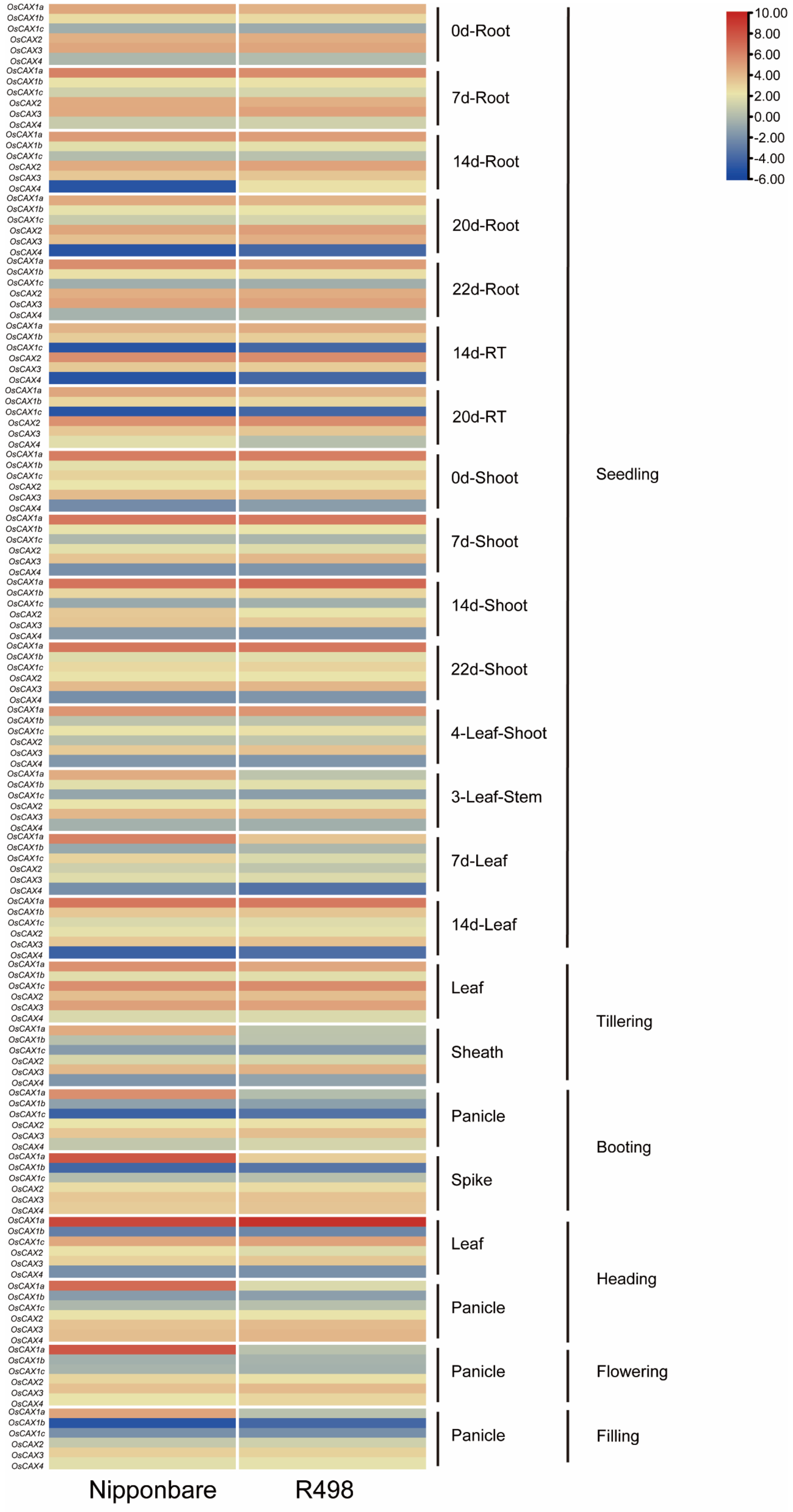

Fig. S6 Comparison of expression profiles of the OsCAX genes in different tissues and developmental stages in Nipponbare (*Geng*) and R498 (*Xian*).

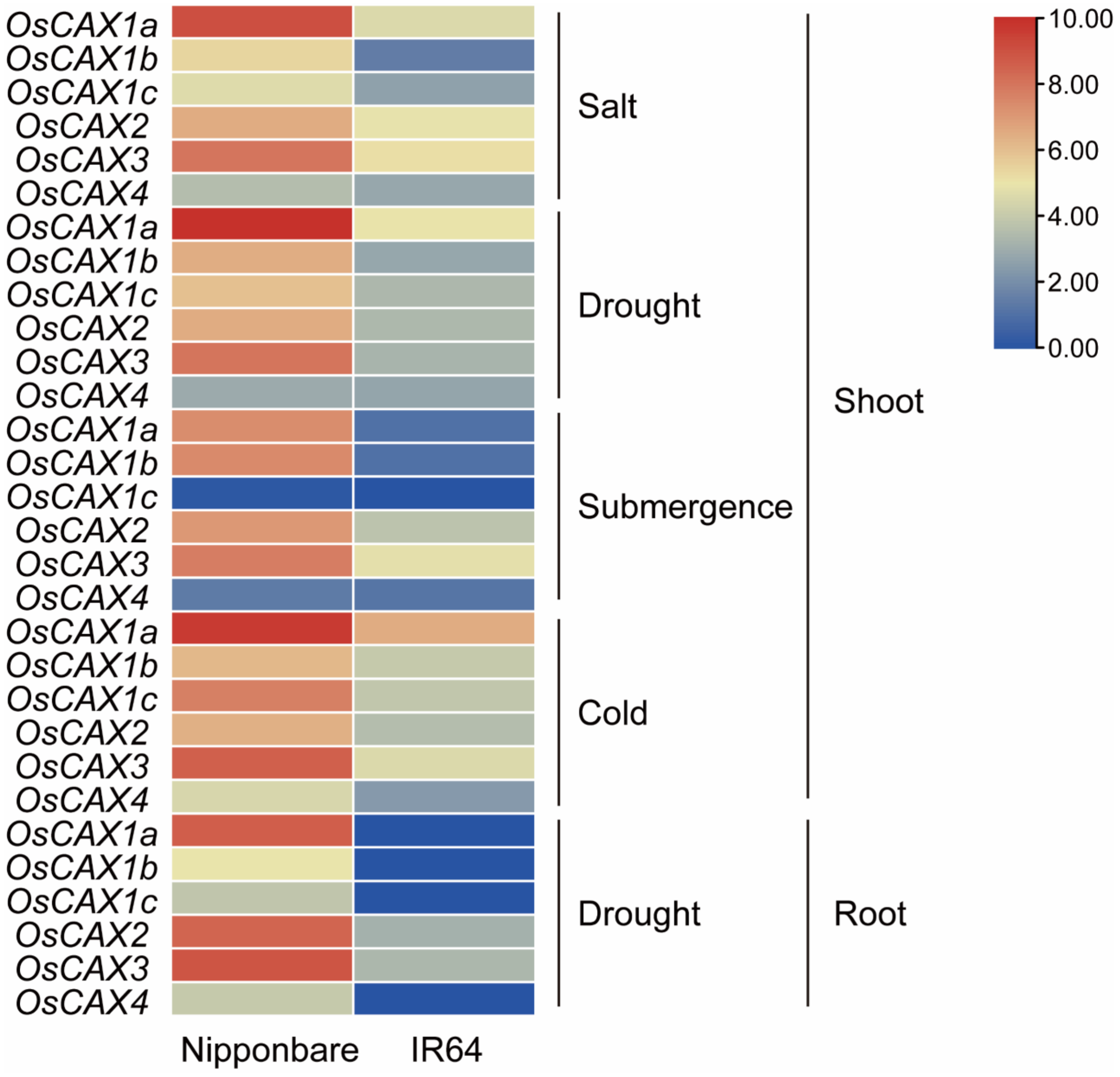

Fig. S7 Comparison of expression profiles of the OsCAX genes in different stress in Nipponbare (*Geng*) and R498 (*Xian)*.

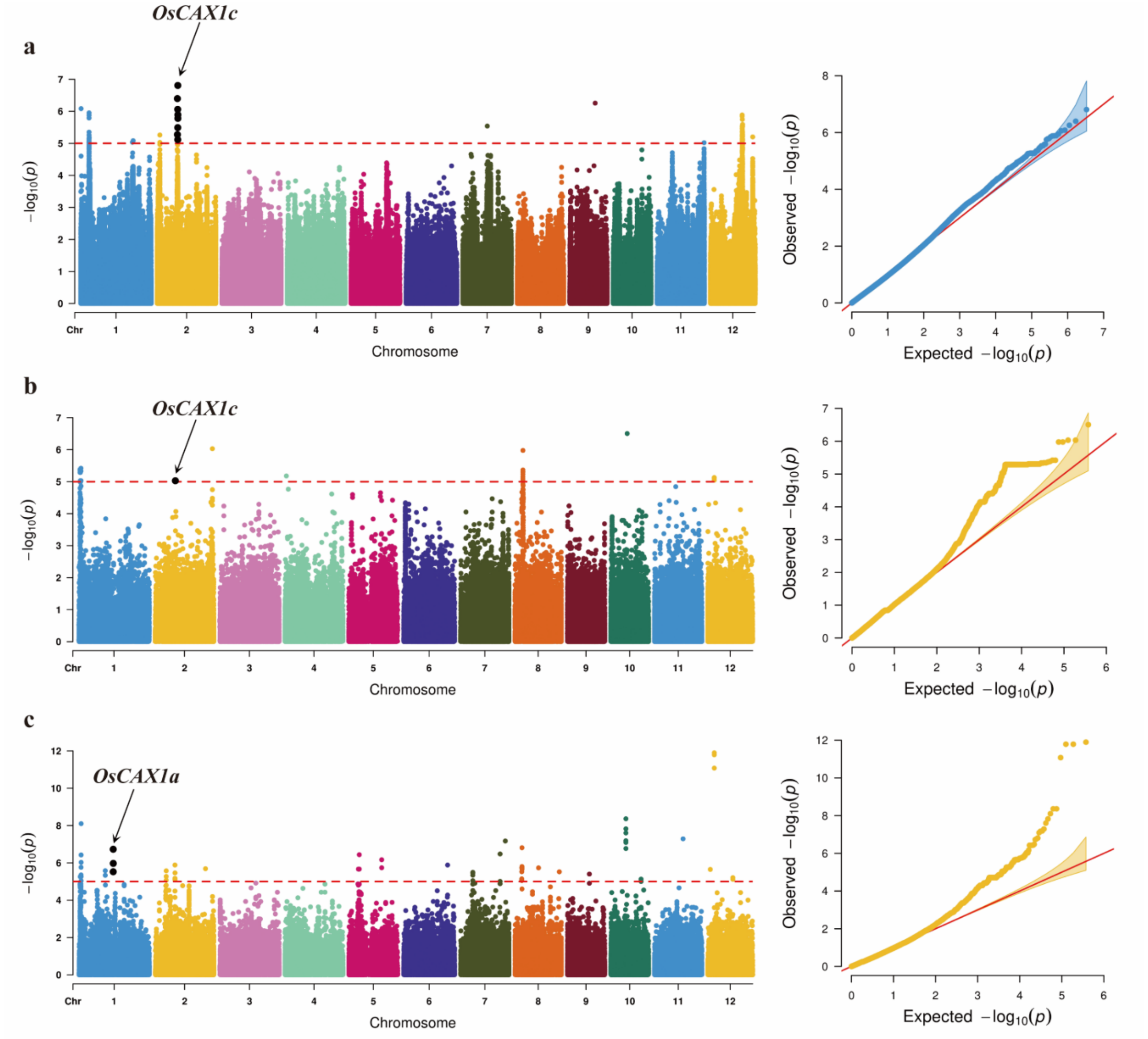

Fig. S8 GWAS of loci associated with germination stage and agronomic traits under salt tolerance in a rice panel, the Manhattan plots and quantile-quantile (Q-Q) plots depicting the GWAS results for three indexs (a. IR48h, b. PL, c. HD) using Mixed Linear Model (MLM). And the horizontal dashed lines indicate the significance threshold of P = 1.0E-5, while the x-axis represents the physical locations across the 12 rice chromosomes, with black points indicating the regions where CAX-genes are identified. IR48h: imbibition rate in 48h, PL: panicle length, HD: days to heading.
